# Diet, Perceived Intestinal Well-Being and Compositions of Fecal Microbiota and Short Chain Fatty Acids in Oat-Using Subjects with Celiac Disease or Gluten Sensitivity

**DOI:** 10.3390/nu12092570

**Published:** 2020-08-25

**Authors:** Lotta Nylund, Salla Hakkola, Leo Lahti, Seppo Salminen, Marko Kalliomäki, Baoru Yang, Kaisa M. Linderborg

**Affiliations:** 1 Food Chemistry and Food Development, Department of Biochemistry, University of Turku, 20520 Turku, Finland; lotta.nylund@utu.fi (L.N.); samahak@utu.fi (S.H.); baoru.yang@utu.fi (B.Y.); 2 Department of Future Technologies, University of Turku, 20520 Turku, Finland; leo.lahti@utu.fi; 3 Functional Foods Forum, University of Turku, 20520 Turku, Finland; seppo.salminen@utu.fi; 4 Department of Pediatrics, University of Turku, 20500 Turku, Finland; marko.kalliomaki@utu.fi; 5 Department of Pediatrics and Adolescent Medicine, Turku University Hospital, 20521 Turku, Finland

**Keywords:** oats, celiac disease, non-celiac gluten sensitivity, intestinal microbiota, gluten-free, SCFAs

## Abstract

A gluten-free diet may result in high fat and low fiber intake and thus lead to unbalanced microbiota. This study characterized fecal microbiota profiles by 16S MiSeq sequencing among oat-using healthy adult subjects (*n* = 14) or adult subjects with celiac disease (CeD) (*n* = 19) or non-celiac gluten sensitivity (NCGS) (*n* = 10). Selected microbial metabolites, self-reported 4d food diaries and perceived gut symptoms were compared. Subjects with NCGS experienced the highest amount of gut symptoms and received more energy from fat and less from carbohydrates than healthy and CeD subjects. Oat consumption resulted in reaching the lower limit of the recommended fiber intake. Frequent consumption of gluten-free pure oats did not result in microbiota dysbiosis in subjects with CeD or NCGS. Thus, the high number of gut symptoms in NCGS subjects was not linked to the microbiota. The proportion of fecal acetate was higher in healthy when compared to NCGS subjects, which may be linked to a higher abundance of *Bifidobacterium* in the control group compared to NCGS and CeD subjects. Propionate, butyrate and ammonia production and β-glucuronidase activity were comparable among the study groups. The results suggest that pure oats have great potential as the basis of a gluten-free diet and warrant further studies in minor microbiota disorders.

## 1. Introduction

Gluten-related disorders form an umbrella for all conditions related to gluten ingestion. These include, most importantly, celiac disease (CeD) and non-celiac gluten sensitivity (NCGS). The prevalence of these disorders has increased over the past 50 years, which makes them emerging health problems worldwide. Celiac disease is a chronic, systemic autoimmune disorder caused by gluten proteins in genetically susceptible individuals. In addition to CeD patients, NCGS subjects also require treatment with a gluten-free diet (GFD). These individuals develop adverse reactions such as gastrointestinal and extra-intestinal symptoms after exposure to gluten [1,2]. A life-long exclusion of gluten from the diet is currently the only effective treatment in alleviating the symptoms of these disorders. The adherence to a GFD and the following recovery from mucosal damage can be assumed to improve the nutritional status of the CeD patients observed at diagnosis. However, a long-term, strict GFD may be challenging to maintain due to social and economic burdens. Even when maintained, GFD may be restricted and nutritionally suboptimal, since many gluten-free products have high fat and sugar but low fiber content. Such a diet predisposes patients to constipation, obesity and cardiovascular diseases [3,4,5].

The use of nutritious and fiber-rich whole-grain oats would diversify the GFD and improve the palatability, texture and fiber-content of the diet. Pure oats are being grown and produced following strict agricultural practices to minimize the contamination with other cereals. In Finland, oats are a major ingredient in the traditional daily diet and since the year 2000, pure oats have been considered suitable for the gluten-free diet [6]. Nowadays, oat products are widely used among Finnish celiac disease patients [7]. Although the inclusion of oats on GFD is recommended in Nordic countries, it is still not globally applied, possibly due to the debate regarding the safety of oats for CeD patients [8,9].

The intestinal microbiota primes the immune system and provides enzymes that expand the metabolic capacity of the host. The conversion of dietary components, such as dietary fiber, that escape the digestion of the host enzymes, support also the growth of microbes themselves. Intestinal microbiota and its metabolites play a major role in defining the antigen milieu of enterocytes, since they are able to interfere with the cells of the intestinal epithelium and modulate the signaling pathways through specific receptors [10]. It is assumed that a decreased microbiota diversity and relative abundances of specific bacterial taxa may lead to functional imbalance where the mutualistic relationship between the host and his microbes is disturbed. Indeed, deviations in the microbiota community structure have been associated with several local and systemic diseases, possibly contributing to the pathogenesis and/or clinical manifestation of these diseases (reviewed in [11]). In addition, GFD as such has been associated with potentially harmful alterations in microbiota, such as decreased microbiota richness, decreased amounts of bifidobacteria, lactobacilli as well as *Faecalibacterium prausnitzii* and increased amounts of *Proteobacteria* [12,13]. However, currently, the majority of the studies published on the fecal microbiota of celiac disease patients have been conducted with pediatric patients or by using conventional methods with limited throughput (reviewed in [14]).

To our understanding, the present study is one of the first on the gastrointestinal well-being and intestinal microbiota of persons with NCGS and within the few evaluating the intestinal microbiota of adult oat-using CeD subjects. The aim of this study was to evaluate the effect of oat consumption on the dietary status and gut well-being among adult subjects with gluten-related disorders who consume oat products on daily basis compared to healthy, oat consuming controls by using fecal microbiota signatures and its metabolites (short-chain fatty acids (SCFAs), ammoniacal nitrogen and β-glucuronidase activity) as biomarkers.

## 2. Materials and Methods

### 2.1. Subjects and Study Design

Celiac disease patients on a remission state (on a GFD at least 1 year), subjects with non-celiac gluten sensitivity (self-reported symptoms occurring after consuming a gluten-containing diet and adherence to a GFD for least 1 year) and healthy controls were recruited to the study. We decided not to test our NCGS subjects according to the Salerno criteria involving a separate gluten challenge trial for reasons discussed in later chapters [15]. The total number of the subjects recruited was 74, of which 49 completed the whole study period. After analyzing the food diary data, 6 subjects from NCGS group were excluded due to the consumption of gluten-containing food products. Thus, samples from celiac disease (CeD) patients (*n* = 19), non-celiac gluten sensitive subjects (*n* = 10) and healthy subjects (*n* = 14) were available for the further analyses. Based on food frequency questionnaire (FFQ) and 4d food diaries, all study subjects reported consumption of oat products daily. Demographic characteristics of study groups are presented in Table 1. Study subjects were recruited to the study from Turku region, Finland during the period August 2017–April 2018. Exclusion criteria were BMI below 18 or above 30, antibiotic treatment within the previous 6 months, use of any medication with gastrointestinal effects (e.g., laxatives or proton pump inhibitors) and blood donation or participation to another clinical study within a month. Before the study entry, the volunteers were interviewed to assess the eligibility of the study. The subjects were ascertained to be in good health by means of self-reporting and normal results in screening blood tests (total blood count, fasting glucose and liver, kidney and thyroid functions, wheatspecific immunoglobulin E (IgE), total immunoglobulin A (IgA) and IgA antibodies to tissue transglutaminase (tTGAbA)). After the screening tests, the study subjects were enrolled in the study and were instructed to keep gut symptom diaries for 30 days. Study subjects consumed their habitual diet throughout the study period and were asked to fulfill food diaries during the last four days of the study. In addition, volunteers were asked to fulfill an FFQ of their dietary habits. Based on the FFQ, The Index of Diet Quality was calculated as explained in detail by Leppälä et al. [16] to assess the adherence to a health-promoting diet. Fecal samples for the microbiota, SCFAs, β-glucuronidase and ammoniacal nitrogen analyses were collected on the last day of the study period. The study protocol was approved by the Ethics Committee of the Hospital District of Southwest Finland (Identifier: ETMK:42/1801/2016) and subjects were enrolled in the study after written informed consent was obtained. The study was registered at ClinicalTrials.gov (Identifier: NCT02761785).

### 2.2. Dietary Intake Using Food Diaries

Subjects were given written and oral instructions on filling the food diaries during the four days preceding the last study visit (including at least 1 weekend day). Kitchen scales were provided to ensure accuracy. Mean daily intakes of energy and macronutrients were calculated by using computerized software (AivoDiet 2.0.2.3; Aivo, Turku, Finland) utilizing the food composition database provided by the Finnish National Institute for Health and Welfare [17].

The quality of overall diet was assessed by FFQ validated for the evaluation of diet quality index [16]. The questionnaire contains 18 questions regarding the frequency and amount of consumption of food products during the preceding week. The quality of the diet was defined as poor when index points were less than 10 out of the maximum 15 points and good when points were 10 or more.

### 2.3. Gut Symptom Diaries

For the 30 day report of perceived gut symptoms, the study subjects were asked to mark down the type of the symptom (upper abdominal pain, lower abdominal pain, cramping, bloating, flatulence, bowel movement, diarrhea or constipation), the severity of the symptom in a scale of 1 to 3 (one meaning mild pain, two being moderate pain and three being intense pain), and the duration of the symptom. The diary was divided into time slots of three hours, except night time, which was marked as six hours slot (from midnight until 6 a.m.).

### 2.4. Fecal Samples and DNA Extraction

Fecal samples were frozen immediately after collection (20 °C) and stored at −70 °C once arrived in the research laboratory which was typically during the defecation day. Microbial DNA was extracted from fecal samples using the repeated bead—beating with KingFisher^®^—method as described in detailed previously [18]. The quality and quantity of the received DNA were measured by using a Nanodrop 1000 spectrophotometer (Thermo Scientific, Wilmington, DE). The quality of the DNA was good in all samples (OD 260/280 ratio ≥ 1.8).

### 2.5. 16S Library Preparation

The library preparation was started from 12.5 ng of total DNA. For NGS (Next-Generation Sequencing) library preparation, the recommended protocol for preparing 16S ribosomal RNA gene amplicons for the Illumina MiSeq system was used (Illumina 2013). The suggested universal bacterial primers were utilized for amplifying the V3 and V4 hypervariable regions of the bacterial 16S rRNA gene with polymerase chain reaction (PCR) using the KAPA Hifi HotStart Ready Mix (Roche Diagnostics Deutschland, Mannheim, Germany). PCR products were purified, and dual indices and Illumina sequencing adapters were attached using the Nextera XT index kit, Illumina. Finally, the libraries were purified once more with AMPure XP beads, Agencourt. The high quality of libraries was ensured using Advanced Analytical Fragment Analyzer and the concentrations of the libraries were quantified with Qubit^®^ Fluorometric Quantitation (Life Technologies, Invitrogen division, Darmstadt, Germany). In a second PCR sample-specific “barcode”—primers and adapter sequences were attached. Up to 96 libraries were normalized and pooled for an Illumina MiSeq sequencing run using the MiSeq Reagent Kit version (v.) 3 with marginally overlapping 300 base pairs (bp) paired-end reads.

### 2.6. 16S rDNA Sequencing

The libraries were normalized and pooled for the automated cluster preparation, which was carried out by Illumina MiSeq instrument. Phix control library was added to the sequencing pool to balance the sequencing run. The libraries were sequenced in a single 2 × 300 bp run with Illumina MiSeq instrument using v3 sequencing chemistry. The sequencing run used paired-end sequencing chemistry with 8 bp dual index run.

### 2.7. Short Chain Fatty Acids Assay

The amounts of fecal short-chain fatty acids (SCFAs) were measured by solid-phase microextraction coupled to gas chromatography and mass spectrometry (SPME-GC-MS) to evaluate the microbial metabolic activity. Fecal samples (0.1 g) were weighted and suspended into 5 mL of deionized water by vortexing. 1.5 mL of fecal suspension was added into 10 mL vial with 0.5 g of NaH_2_PO_4_ [19]. Acetic acid, propanoic acid and butyric acid (Sigma-Aldrich, WGK Germany) were used as external standards in order to control the daily variation of instrument and sample preparation. The SPME fiber used was 75 µm CAR/PDMS, Fused Silica (Supelco, Bellefonte, PA, USA). The SPME-GC-MS analysis was carried out with Thermo Trace 1310—TSQ 8000 Evo equipped with an autosampler (Thermo Scientific, Wilmington, DE, USA). Compounds were separated by Supelco fused silica capillary column SPB-624, (30 m × 0.25 mm × 1.4 µm) under a carrier gas (helium) 1 mL/min with a splitless mode. The oven temperature program was as follows: 40 °C hold for 2 min and then 5 °C/min rise until 200 °C, hold for 10 min. A voltage of 70 eV was set in the EI. The system was operated using Xcalibur 4.0 (Thermo Scientific, Wilmington, DE, USA). Compounds were identified by the NIST library [20] and quantified by comparison to external standards. To optimize the SPME analysis, five commercial fibers were screened: 50/30 µm DVB/CAR/PDMS, 65 µm PDMS/DVB Stableflex, 65 µm PDMS/DVB Fused Silica, 100 µm PDMS and 75 µm CAR/PDMS. 75 µm CAR/PDMS was evaluated by comparison of SCFA standard runs as the most suitable for SCFA detection and chosen for the analysis.

### 2.8. β-Glucuronidase and Ammoniacal Nitrogen Assays

The activity of β-glucuronidase enzyme and the amount of ammoniacal nitrogen were measured from fecal samples to evaluate differences in these potentially harmful microbial metabolic activities. Ammoniacal nitrogen assay was carried out by an indophenol blue method reported in detail elsewhere [21]. Briefly, 0.1 g of wet fecal sample was diluted with 5 mL of deionized water, shaken for 60 min, and centrifuged at 3000× *g* for 3 min. Ammoniacal nitrogen concentration was measured from supernatant based on absorbance measured at 630 nm (Hidex Sense microplate reader, Hidex Oy, Turku, Finland). β-glucuronidase assay was carried out by the protocol of Shen [22]. Briefly, 0.1 g of wet fecal sample was diluted with 5 mL of deionized water and shaken for 60 min. 0.1 mL of diluted sample was added into Eppendorf tube^®^ with 0.4 mL of 2 mM *p*-nitrophenyl-β-d-glucuronide solution (Sigma Aldrich, WGK Germany). Suspensions were incubated in anaerobic conditions at 37 °C for 60 min, followed by addition of 0.5 mL of 0.5 M NaOH. This suspension was centrifuged at 3200× *g* for 10 min and absorbance was measured on 405 nm (Hidex Sense microplate reader, Hidex Oy, Turku, Finland).

### 2.9. Statistical and Data Analyses

Statistical analyses of food diary and microbial metabolites data were carried out using IBM SPSS Statistics 25 software. Normal distribution of data was tested with Shapiro–Wilks test and ANOVA with contrast test were used to determine the statistical differences between study groups.

In the preprocessing of the MiSeq sequencing reads, the workflow proposed by [23] was adapted. In summary, the reads were trimmed from the left at 25 bp and 10 bp for the forward and reverse reads, respectively; and from right at 245 bp and 230 bp based on manual inspection of the read quality summaries. The sequence variant table was constructed from the reads with DADA2 [24] based on DADA2-formatted training FASTA files that were derived from the Ribosomal Database Project’s Training Set 16 and the 11.5 release of the RDP database [25]. The chimeras were removed. The phylogenetic tree was constructed with the DECIPHER [26] and phangorn R packages. The preprocessed data were converted into a phyloseq R object [27], and aggregated to the genus level with the microbiome R package (function aggregate_taxa). The full details are available in the source code that is openly deposited at Zenodo.

The Principal Coordinates Analysis (PCoA) was done for compositional data based on Bray–Curtis dissimilarity. Alpha diversity (Shannon index) was estimated with the *microbiome* [28] and *vegan* [29] R packages. For standard data manipulation and visualization, the *tidyverse* and *ggplot2* R packages were used, respectively. Beta diversity was done with PERMANOVA using the vegan R package and 999 permutations. The analyses were done with genus-level clr-transformed abundance tables unless otherwise mentioned. The group-level comparisons for individual genera were done with DESeq2.

## 3. Results

### 3.1. Dietary Intake and the Quality of Diet

Based on the food diary data (4 days), NCGS subjects received a higher proportion of their energy (E %) from fat and lower proportion (E %) from carbohydrates when compared to healthy controls (*p* = 0.025 and *p* = 0.045, respectively) (Table 1). Additionally, the gluten-sensitive subjects tended to get more energy than celiac disease patients when adjusted per body weight (kcal/kg of body weight, *p* = 0.09, data not shown). The mean intake of dietary fiber was at the lower end of the recommendation level in the three groups (Table 1). The dietary quality assessed by the validated index of diet quality questionnaire was considered good in most of the study subjects, average diet quality indices being higher than 10 in most of the study subjects (Table 1).

### 3.2. Gut Symptom Diaries

The highest amounts of gut symptoms per subject was reported by the NCGS groups subjects (61.4) when compared to CeD and healthy controls (39.1 and 19.7, respectively) (*p* = 0.045). In all study groups, the most often reported symptoms were flatulence, bloating and lower abdominal pain.

### 3.3. Intestinal Microbiota Signatures

**Figure 1:**
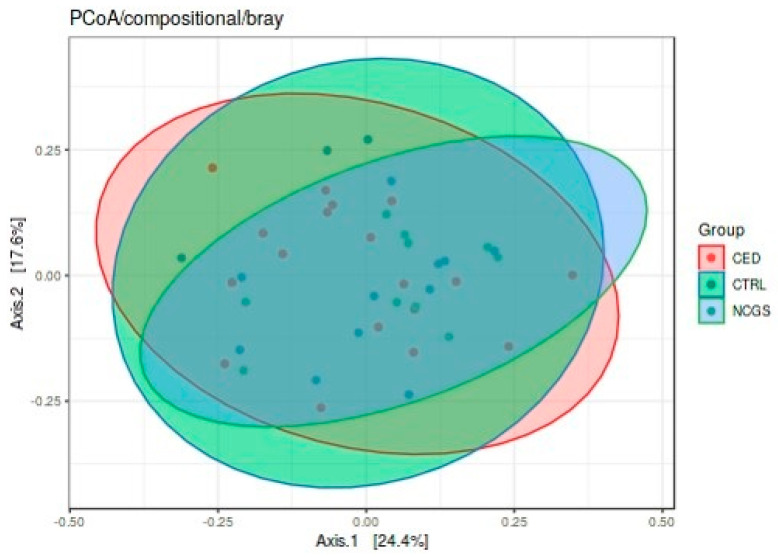
Total microbiota profiles of study subjects were comparable between the groups as assessed by Principal Component Analysis (PCoA). CED celiac disease (*n* = 19), NCGS non-celiac gluten sensitivity (*n* = 10) and CTRL healthy controls (*n* = 14).

**Figure 2:**
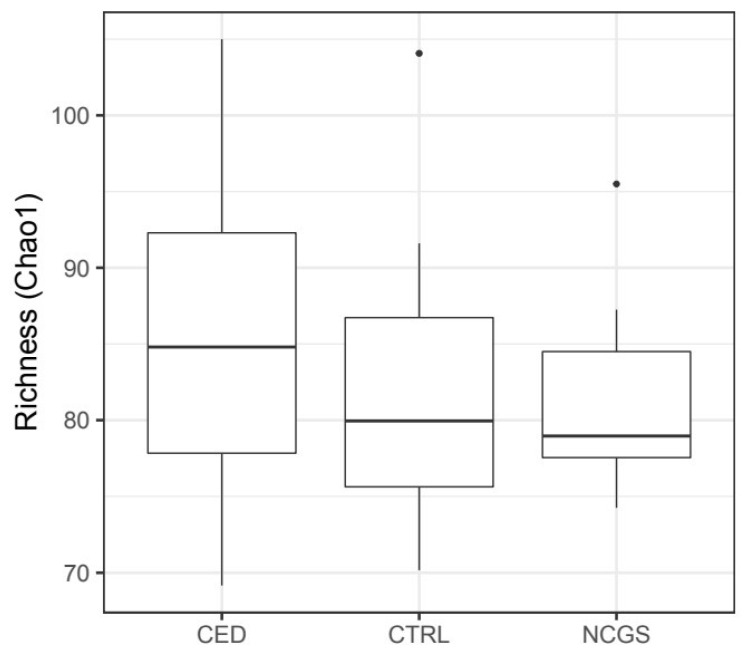
Microbiota richness was comparable in subjects with celiac disease (CED) (*n* = 19), non-celiac gluten sensitivity (NCGS) (*n* = 10) and healthy controls (CTRL) (*n* = 14). The box extends from 25th percentile to 75th percentile, with a line at median.

**Figure 3:**
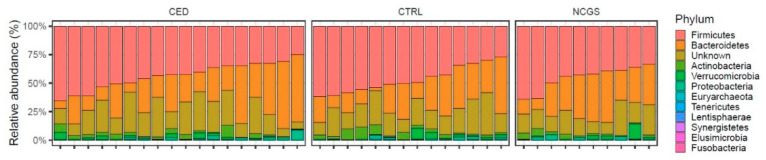
Relative abundances of bacterial phyla (% of total reads) in subjects with celiac disease (CED) (*n* = 19), healthy controls (CTRL) (*n* = 14) and subjects with non-celiac gluten sensitivity (NCGS) (*n* = 10). No statistically significant differences were observed between the study groups.

**Figure 4:**
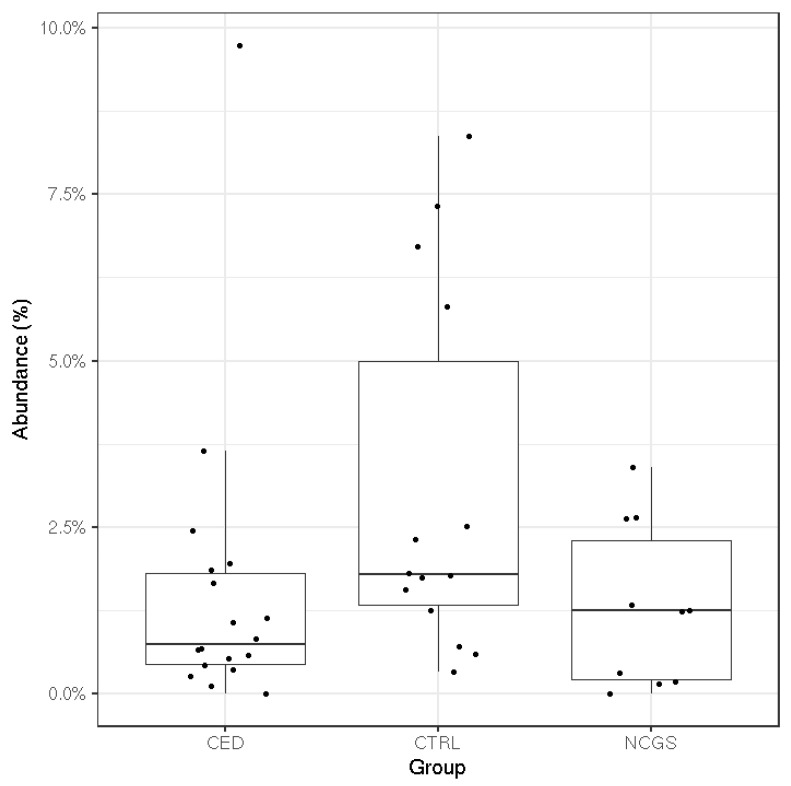
The mean relative abundance of *Bifidobacterium* in subjects with celiac disease (CED) (*n* = 19), healthy controls (CTRL) (*n* = 14) and subjects with non-celiac gluten sensitivity (NCGS) (*n* = 10). The difference between the three was borderline significant (*p* = 0.067; Kruskal–Wallis test).

The total microbiota profiles were comparable between CeD, NCGS and healthy controls (Figure 1). No statistically significant differences were observed in microbiota richness (Figure 2) or diversity (data not shown) between the study groups. Phylum-level microbial abundances were characterized by a high inter-individual variation and no statistically significant differences were observed between the study groups (Figure 3). However, the abundance of *Bifidobacterium* tended to be higher in the control group compared to CeD and NCGS (*p* = 0.067), (Figure 4).

### 3.4. Microbial Metabolic Activity

In CeD subjects, the amount of SCFAs was comparable to other groups (Table 2). However, the relative amount of acetate (% of total SCFAs) was higher in the control group compared to the NCGS group (*p* = 0.03). No statistically significant differences were observed in the proportions of propionate or butyrate between the groups. In addition, the amounts of ammoniacal nitrogen and β-glucuronidase activity were comparable between the study groups (Table 2).

## 4. Discussion

Currently, the only treatment for celiac disease and other gluten-related disorders is a life-long adherence to a GFD. Due to the shortage of whole-grain products in the diet, GFD often results in inadequate intake of nutrients and dietary fiber [30,31] while in our study the average intake of dietary fiber intake was at the lower end of recommendation in all three groups, and did not differ between them. The difference may result from the fact that our subjects consumed oat products as a part of their habitual diet. Pure oats suitable for the gluten-free diet are grown, milled and handled without contamination by other cereals. Recently, it was suggested that the current confounding clinical findings on the safety of oat consumption in CeD subjects [32] could be caused by contaminated oats assessed as “pure” [8]. Indeed, a decade ago, gluten cross-contamination was shown to exist in oat supply chains in Europe, the United States and Canada [33]. Yet, our results suggest a great potential for pure oats as a source of fiber to GFD.

While persons diagnosed with CeD receive professional dietary advice in Finland (The Finnish Medical Society Duodecim 2018), many NCGS subjects are self-educated in GFD. In our study, the dietary composition of CeD subjects was comparable with healthy controls while subjects with NCGS obtained more energy from fat (>40 E %) and less energy from carbohydrates when compared to healthy controls. In addition, the food diaries in this study revealed that a large number of volunteers initially assigned to the NCGS group (6/16) consumed gluten, most often from rye or barley. This may be an indication that they are not aware of the composition of GFD. According to a recent survey study of Potter et al. [34], 24% of responded Australians avoided gluten completely or partially, while 14% had self-reported non celiac wheat sensitivity and 1% had celiac disease. Others avoided gluten for “general health” or as a treatment of abdominal pain, without being diagnosed with CeD or NCGS. The authors considered that gluten avoidance may be due to the current well-being trend and that NCGS may overlap with other gastrointestinal disorders [34]. Additionally, we observed that the NCGS group reported more gut symptoms per subjects when compared to CeD and healthy controls (*p* = 0.045). This finding is in line with a recent study by Tovoli et al. [35], where a significant proportion (66%) of gluten-sensitive subjects, diagnosed according to Salerno criteria, reported intestinal symptoms even years after the beginning of GFD. Compared to CeD patients following the same diet, subjects with NCGS reported a higher amount of symptoms (33%). Additionally, Skodje et al. (2019) reported a high number of gastrointestinal complaints among subjects with self-reported NCGS on a GFD.

The existence of NCGS as a condition has been recently challenged and intake of other non-gluten wheat components such as fructans [36] and amylase–trypsin inhibitors have been suggested to lie behind the symptoms instead of gluten as such [37]. Even a term change from NCGS to non-celiac wheat sensitivity has been suggested [38]. Still, NCGS has its defenders among consumers and researchers. The Salerno criteria [15] have been proposed for standardization of the diagnosis of NCGS. Criteria based investigation involves reporting of symptoms during 6 weeks on gluten-containing diet followed by 6 weeks of GFD and a further 1 week of test period containing GFD supplemented with either gluten test meals or placebo, 1-week washout and another test period in a cross-over manner. For the status of NCGS, 30% variation of symptoms between GFD and gluten containing diet periods is required. Such a gluten challenge was not imposed on the NCGS subjects in our trial due to limited resources and burden on the volunteers to participate on multiple clinical investigations. More so, our aim of this study was not to investigate which proportion of our self-reported NCGS volunteers would fulfil the Salerno criteria nor to limit our volunteers only to those getting gastrointestinal problems in the gluten challenge but to include subjects who self-reported their need for gluten-free diet despite lack of diagnosis for CeD or wheat allergy. Instead, the subjects were screened for negative celiac serology, specific immunoglobulin E (IgE) and wheat allergy. Generally, CeD patients are screened to ensure the remission state of their disease and NCGS patients to exclude the celiac disease before landing on NCGS diagnosis. However, once the GFD is initiated, testing for celiac disease is no longer accurate, which may lead to false-negative results in the case of self-diagnosed NCGS patients.

Previously, the majority of the studies analyzing the fecal microbiota of celiac disease patients have been conducted with pediatric patients or by using conventional methods with limited throughput [14]. The most often reported hallmarks of CeD microbiota have been increased abundances of Gram-negative bacteria, such as *Proteobacteria* and *Bacteroidetes* and reduced abundances of *Bifidobacterium* spp. and *Lactobacillus* spp. [14]. Similar changes have been also reported in studies examining the microbiota of healthy subjects after 1 month on GFD [12,13]. Moreover, some studies have reported persistent microbiota dysbiosis in CeD subjects in remission and on a GFD [39,40,41]. Of these studies [12,13,39,40,41], only Wacklin et al. (2014) report that the subjects consumed oats. Likewise, the studies reviewed by Marasco et al. (2016) concerning the microbiota composition of CeD patients or subjects following GFD, do not report oat consumption of the subjects, apart from the mentioned study of Wacklin et al. (2014). Our study on pure-oat consuming CeD subjects did not detect any signs of microbiota dysbiosis typically observed in CeD subjects with active disease nor detected any major GFD related changes in microbiota on NCGS or CeD subjects. The mean abundance of *Bifidobacterium* was higher in the control group compared to CeD and NCGS subjects but the difference was only marginally significant (*p* = 0.067). One of the CeD subjects had a high level of *Bifidobacterium* (9.7%). The abundance is within the typical range of variation for this genus, although it was unexpected to observe in the CeD group which has been associated with a reduced level of *Bifidobacterium*. This, combined with the moderate sample size of our study, may partially explain the only marginally significant difference between the groups. Therefore, our results do not support the hypothesis that a significant intestinal microbiota dysbiosis would be the reason for the increased gastrointestinal symptoms reported by the NCGS group. The obtained results of microbiota composition agree with another Finnish study, where the CeD status of children who had consumed pure oats for 2 years was evaluated [42]. Small intestinal biopsies of these children showed normal histology and they had normal serological markers. Follow-up was continued for 7 years and all the markers remained normal during this period, suggesting that oats were well tolerated [42].

SCFAs are an important energy source for enterocytes and have been associated with several health-promoting effects including antipathogenic effects [43,44]. Their production varies among the individual microbiota compositions and by the type and amount of carbohydrates consumed [45]. We found that the relative amount of acetate (% of total SCFAs) was higher in the control group compared to NCGS group (*p* = 0.03), which may be linked to the higher abundance of *Bifidobacterium* in the control group compared to CeD and NCGS groups (*p* = 0.067) [46]. Proportions of propionate or butyrate did not differ between the groups. Thus, the intestinal microbiota of oat-using CeD and NCGS subjects was capable of producing similar amounts of propionate and butyrate than that of healthy controls. Previously, clinical studies assessing the SCFA levels have focused mainly on healthy adults [47,48,49], whereas adult celiac disease patients in remission have not been studied so far. Di Cagno et al. [50] analyzed volatile compounds from fecal samples of treated CeD children by SPME-GC-MS. The samples showed lower levels of SCFAs, such as butyric, isocaproic, and propanoic acids, when compared to healthy controls. However, the dietary habits of their subjects were not reported. It should be noted that SCFAs are rapidly absorbed in the colon and thus the fecal SCFA reflects losses rather than the amount of production in situ [43]. However, access to the proximal colon to quantify SCFA production rates is invasive and not possible in most study settings. Therefore, measurement of the fecal SCFAs is currently the only feasible way to estimate the production of compounds by gut microbiota. In this study, the interindividual variation of free-living human volunteers was large, but within the range observed previously [47,48,49]. The variation could possibly have been influenced by restrictions on the other parts of the diet than oats, but such were not applied in this study.

β-glucuronidase enzymes expressed by the intestinal microbiota mediate the reactivation of molecules important in human health and disease. For example, microbial glucuronidases regenerate toxic carcinogens whose increased activities in the GI tract have been associated with a higher incidence of gastrointestinal diseases such as colon cancer, Crohn’s disease and colitis as well as to high-fat diets [51]. In rodent studies, high consumption of dietary fiber has been associated with decreased activity of β-glucuronidase [22,52]. In addition, a clinical crossover trial with 28 overweight male subjects demonstrated significantly decreased β-glucuronidase activity after consumption of wholegrain wheat and rye, when compared to low fiber control diet [53]. Our results show that CeD and NCGS subjects, who consume oat products on a daily basis, have similar β-glucuronidase activity levels than healthy controls.

No differences were seen in the ammonia production among the study groups. Ammoniacal nitrogen is a microbial end product produced by the deamination of amino acids. It is harmful to the host in high amounts, and previously the consumption of dietary fiber has been detected to decrease its production [53,54,55]. A study comparing the impact of diet on in vitro fermentation properties of whole grain flours and brans from corn, oats, rye and wheat reported significantly reduced ammonia concentrations after fermentation of oats and rye when compared to corn or wheat [54]. In general, ammonia concentrations of all groups studied were in line with previous results measured from healthy adults [47,56,57,58,59,60].

The strengths of this study were accurate analyses of fecal microbiota composition and metabolites of adult CeD and NCGS subjects, which has only received limited attention. A unique strength of the study was also the comparison of biological data to perceived symptoms by utilizing the gut symptom diaries. Moreover, the reliability of the results increased/enhanced the understanding of individual dietary habits of study subjects by analyzing 4d-food diaries as well as food frequency questionnaires assessing the overall quality of the study subject’s diet. Additionally, the suitability of the study subjects was ensured by a detailed interview and screening at the recruitment to this study. The lack of non-oat-using CeD subjects can be considered as a limitation of this study since the inclusion of these subjects would have enabled a more accurate evaluation of the effect of the oat consumption on microbial biomarkers in celiac disease. However, the recruitment of non-oat using CeD subjects for the current study proved to be impossible. In Finland, the consumption of oats has been allowed for adult CeD subjects since 1997 and for pediatric patients since 2000 [6,61], and currently, most of the Finnish CeD subjects consume oats as part of their GFD [6,61].

To conclude, this study evaluated the influence of daily pure oat consumption on perceived and measured gut well-being in adult subjects with celiac disease and with subjects with non-celiac gluten sensitivity compared to healthy volunteers. No microbiota dysbiosis was detected among CeD nor NCGS subjects. However, further studies with metagenomic approaches should be conducted to assess the potential differences in microbiota composition and function in celiac patients consuming oats. The results of this study suggest that pure oats in a gluten-free diet represent a good alternative. We also demonstrate the need for further studies in NCGS subjects focusing on diet and microbiota interactions, and nutrition counseling to identify the causes of perceived gut symptoms.

## Figures and Tables

**Table 1:** Basic characteristics and dietary intake of study subjects.

GROUP	CeD (*n* = 19)	NCGS (*n* = 10)	CTRL (*n* = 14)	*p*-Value
Subjects (*n*)	19	10	14	n.s.
Male/Female ^1^	4/15	1/9	6/9	n.s.
Age (year) ^2^	51 (24, 65) ^a^	34 (22, 61) ^b^	34 (24, 63) ^b^	0.020
BMI (kg/m^2^)	24.6 (3.2)	23.0 (2.6)	24.4 (2.6)	n.s.
Proteins (E %)	17.1 (3.6)	16.5 (3.4)	15.8 (3.1)	n.s.
Carbohydrates (E %)	41.9 (4.9) ^a,b^	40.3 (6.1) ^a^	45.8 (4.8) ^b^	0.045
Fat (E %)	36.4 (5.7) ^a,b^	41.0 (6.2) ^a^	34.7 (4.6) ^b^	0.025
Dietary fiber (g)	25.5 (9.1)	27.6 (7.7)	26.0 (7.4)	n.s.
Saccharose (g)	46.2 (19.3)	40.8 (12.3)	52.5 (23.0)	n.s.
Diet Quality Index	10.9 (1.7)	10.2 (2.2)	10.3 (1.5)	n.s.

Dietary data are presented as an average of 4d intake based on food diaries. Values are mean (SD), unless otherwise stated. CeD subjects with celiac disease, NCGS non-celiac gluten sensitivity, CTRL healthy controls. ^1^ Pearson Chi-Square. Others One-way ANOVA. ^2^ median (min, max) Values with different letters differ from one another in each row.

**Table 2:** Production of short-chain fatty acids (SCFAs), ammonia and the activity of β-glucuronidase in subjects with celiac disease (CeD), non-celiac gluten sensitivity (NCGS) and healthy controls (CTRL).

	CeD (*n* = 19)		NCGS (*n* = 10)		CTRL (*n* = 14)	
	Concentration	% of Total SCFA	Concentration	% of Total SCFA	Concentration	% of Total SCFA
Fecal acetic acid (µg)	2144 (1228)	63 ^a,b^	2149 (1205)	59 ^a^	2789 (1473)	71 ^b^
Fecal propionic acid (µg)	806 (607)	23	948 (451)	28	698 (521)	19
Fecal butyric acid (µg)	337 (128)	14	456 (258)	13	424 (327)	10
Total SCFA (µg)	3287 (1786)		3553 (1680)		3912 (2072)	
Fecal ammonia (µmol)	18.0 (6.5)		18.5 (4.8)		15.7 (7.2)	
Fecal β-glucuronidase (U)	30.0 (15.0)		25.9 (15.0)		29.9 (18.0)	

Concentrations are presented per g of fecal wet weight. Values are presented as mean (SD). Values with different letters differ from one another in each row.

## Data Availability

The cohort datasets generated and/or analyzed during the current study are not publicly available due to confidentiality, to protect the cohort participants’ identity. The microbiota dataset analyzed in the current study is available from the corresponding author on reasonable request.
